# Ultra Brief Psychological Interventions for COVID-19 Pandemic: Introduction of a Locally-Adapted Brief Intervention for Mental Health and Psychosocial Support Service

**DOI:** 10.21315/mjms2020.27.2.6

**Published:** 2020-04-30

**Authors:** Nicholas Pang Tze Ping, Wendy Diana Shoesmith, Sandi James, Noor Melissa Nor Hadi, Eugene Koh Boon Yau, Loo Jiann Lin

**Affiliations:** 1 Faculty of Medicine and Health Sciences, Universiti Malaysia Sabah, Sabah, Malaysia; 2 Department of Social Work and Social Policy, School of Science, Health and Engineering, La Trobe University, Australia; 3 Department of Psychiatry and Mental Health, Hospital Tuanku Fauziah, Perlis, Malaysia; 4 Department of Psychiatry, Faculty of Medicine and Health Sciences, Universiti Putra Malaysia, Selangor, Malaysia; 5 Queensway Clinic, Central and North West London NHS Foundation Trust, Milton Keynes, United Kingdom

**Keywords:** psychotherapy, mental health, COVID-19, psychiatry, PFA

## Abstract

The ultra-brief psychological interventions (UBPI) was created in 2018 to empower healthcare providers with psychological skills that can be delivered within a short period. Techniques used within UBPI were adopted from a variety of well established psychotherapies and distilled into its core essentials. This enabled practitioners of UBPI to deliver specific psychological skills in the appropriate context to the client within a period of 15–20 min. UBPI was also manualised to standardised training of practitioners. During the novel coronavirus disease of 2019 (COVID-19) pandemic, UBPI was modified to suit the unique psychological demands of the pandemic. This article presents how UBPI was adapted and used with healthcare providers dealing with COVID-19 and also with the public who required psychological first aid (PFA).

## Introduction

The novel coronavirus disease of 2019, otherwise known as COVID-19, was declared as a global pandemic with an alarmingly fast spread and worrisome consequences by the World Health Organization (WHO) during a media briefing in March 2020. Four key points were highlighted: i) be prepared for the pandemic; ii) focus on detecting, protecting and treating those affected; iii) reduce transmission and iv) innovate and learn. These recommendations involved multiple stakeholders, from frontline healthcare, public health and even political leaders. These recommended measures have thrown up unique challenges to various countries due to limitations in capacity, resources or resolve, including Malaysia (1).

Mental health is a crucial area that cannot be ignored during this critical period. The multiple changes brought upon us all by the pandemic pose a unique challenge in mental health service delivery. The restriction in freedom of movement and face to face service provision are examples of new challenges. As a result, there needs to be a dramatic shift in how psychological services are to be delivered. Li et al. (2) summarises how China is addressing these challenges with a progressive and dynamic approach. One of the main adaptations was the creation of emergency psychological crisis intervention services and the formation of expert groups that can provide professional input with health authorities.

There are also new barriers in mental health delivery during this COVID-19 crisis in Malaysia. The strict movement restriction order and isolation procedures that were implemented, while necessary, have been observed to be generating an increasing level of psychological distress amongst the general population. While evidence of current levels of psychological distress within the general population of Malaysia is not available, Qiu et al. (3) reported that a nationwide survey in China revealed that at least one-third of its participants reported psychological distress. Healthcare providers, especially those on the frontlines, are also very likely to encounter increased levels of stress. The stress puts them in a vulnerable state where they are at increased risk of developing psychological distress, vicarious trauma or acute stress disorder. The stress also has the potential to develop into a pathological illness, e.g. post-traumatic stress disorder, anxiety and depressive disorders. There are no current publications available regarding psychological distress in healthcare providers, but there is at least one report from China regarding healthcare providers experiencing psychological distress (4). The use of a psychological intervention that addresses these issues is warranted. Zhang et al. (5) suggested that any intervention during the ongoing period of COVID-19 pandemic should be focused on imparting rapid adaptation skills and also psychological first aid (PFA). As such, the authors would like to present here an ultra-brief psychological interventions (UBPI) module that is adapted specifically to the COVID-19 pandemic.

## UBPI

Created by authors Shoesmith and James, UBPI training was introduced in Universiti Malaysia Sabah (UMS) in 2018. UBPI was manualised in the form of a handbook (6) and used in conjunction with the training. The training was provided in various psychiatric settings, involving doctors, nurses, psychologists, speech therapists and counsellors from both primary and specialised care settings. It allows for various psychological interventions, taking less than 15 min, to be performed by health professionals. The UBPI skills incorporate multiple techniques from various evidence-based psychological interventions, including cognitive-behavioural therapy (CBT), acceptance and commitment therapy (ACT), dialectical-behavioural therapy (DBT), motivational interviewing (MI) and early intervention programme (EIP). They also include collaborative skills, such as team-based problem solving, shared decision making and validation skills (Table 1). This course was started as part of an action research project to improve collaborative practice in the Malaysian mental health system. Qualitative research had shown that the system was frequently not working collaboratively, in that patients were not forming a therapeutic alliance with any of their healthcare providers and a hierarchical culture meant that staff were not collaborating well with each other (7). The techniques are small discrete micro-skills that have been extracted from the several psychotherapies and simplified for delivery. Some of the skills are also translated into Malay for practitioners to be able to use effectively in the Malaysian context. Although large scale quasi-experimental studies are still in the pipeline, there was positive qualitative feedback from different practitioners, with reports of ongoing use of UBPI in the clinical setting. A case series on how UBPI could be used was presented in the 1st International Meeting of the World Psychiatric Association Psychotherapy Section (Koh et al.) (8).

To address the current COVID-19 pandemic, UBPI was modified to tailor to the psychological needs that have been observed to accompany the pandemic and subsequently compiled into a manual (Nor Hadi et al.) (9). The UBPI for COVID-19 (UC-19) self-guided manual was created in the Malay language to cater to as many users in Malaysia as possible. Skills and techniques adopted include the following: supportive aspects and general communication skills e.g. empathy, validation and reflection, mindfulness techniques, anchoring, physicalising, shared decision making, problem solving and brainstorming techniques. The manual emphasises short and practical interventions that can be performed by most health and social service staff, even without a psychological background.

This project could have extensive public health implications by equipping relevant stakeholders in basic psychological skills that are essential for managing the impact of COVID-19. This paper details attempts to deploy the UC-19 in two unique scenarios: occupational mental health and PFA.

## UBPI for Occupational Mental Health in COVID-19

The initial use of UC-19 was a trial with 25 Hospital Universiti Malaysia Sabah (HUMS) nurses who were working on the COVID-19 frontline in early February 2020. These nurses have been employing these techniques for a month and have been giving positive qualitative feedback regarding them. Subsequently, the feedback was adopted and UC-19 was created as a response to the urgent need for large-scale ongoing preventive and intervention measures. UC-19 contained specific examples taken from on the ground HUMS frontliners for COVID-19. This made it more relatable to the staff who were having COVID-19 related anxiety on the ground. The course put a heavy emphasis on validation skills, which were adapted from the DBT treatment manual (10). Participants were also trained in giving feedback to others, including people higher than them in the hierarchy. The course also emphasised values and goals, adapted from ACT, where participants were asked to consider their own values as well as team values. Participants were taught group problem solving techniques and asked to solve real world problems facing the clinic at that time. Mindfulness skills and how to apply this to both individual and team reflective practice were also taught. A manual was created, which also included other skills, such as emotional regulation skills from DBT. The manual was piloted for a week in Hospital Tuanku Fauziah, Perlis, Malaysia and then shared to various centres nationwide by both mental health workers, frontliners and the general public. It was also distributed via www.actmalaysia.com to increase availability.

So far, informal qualitative feedback has suggested that it has helped respond effectively to individuals with anger or frustration, anxiety secondary to the uncertainty of the daily fluctuations of COVID-19, panic and tension in committee meetings, and the general psychological wellness of hospital staff. Participants found the validation skills particularly helpful and several described how team communication had changed after the course, in that people were more open and less judgemental.

## UBPI for PFA for Public in COVID-19

The UC-19 was also used to provide PFA with the general public. A web-based platform was set up jointly by Hospital Mesra Bukit Padang, the tertiary referral psychiatric hospital in Sabah, Malaysia and the UMS mental health team. The platform, called COVIDCare, is a web chat portal to provide PFA to anyone who experiences anxiety or psychological distress due to COVID-19. On top of the generic PFA principles to ‘look, listen and link’, principles of UBPI were used to upskill the PFA frontliners. Daily virtual group supervision was conducted as part of the effort of quality control on competency.

Feedback from users of the platform, either as a client or provider, had thus far been positive. The online delivery method is particularly useful as students in UMS are not allowed to leave the campus during the movement restriction order. The students are experiencing increasing levels of anxiety related to concerns of not having enough food and other essential supplies to worries about contracting the illness. UBPI principles were used by the providers to help students to remain grounded and be in the present moment instead of constantly worrying about the future or ruminating about the past. The providers also guide the students in problem solving when overwhelming anxiety prevents them from responding effectively. Validation and reassurance about the distress that they are currently undergoing was also offered.

In order to optimise available human resources and skills, UC-19 skills were task-shifted to pre-clinical medical students while the doctors focus on other clinical matters. The students were able to manage the PFA hotlines and provide the necessary brief psychological interventions after a short training. The students also received real time supervision while they provide PFA services. All training, supervision and services provided were done online and social distancing was maintained. This highlights the strength of UBPI: the ease of adoption, training and use meant that it could be rolled out to other centres quickly and rapidly. All of that can also be done online, thus removing geographical limitations for trainers, providers and clients.

Some of the module contents have also been adopted by a non-profit organisation, Pertubuhan Kesejahteraan Psikospiritual Malaysia, on their social media platform. They used the module’s content to provide information regarding self-management of mental health during the pandemic. While not part of the UBPI initial objectives, this reflects the module adaptability in illness self management where a person is enabled and trained to self-regulate and manage his or her health.

The online delivery of UC-19, i.e. telepsychiatry, does warrant discussion on the effectiveness as a medium, its limitations and ethical considerations. Telepsychiatry is defined as ‘the delivery of healthcare and the exchange of healthcare information for the purposes of providing psychiatric services across distances’ (Wootton et al.) (11). While the use of telepsychiatry is both efficacious and well-received, there is an important ethical consideration regarding confidentiality and risk management (12). It is possible for telepsychiatry consultations to be observed by outsiders, with or without the client’s knowledge (12). Thus, breaching confidentiality. Also, the delivery of UC-19 via phone call limits the availability of essential cues, i.e. non-verbal cues, that can help the provider in service delivery. As such, informed consent from clients of COVIDCare is required before starting. They will be informed that they will only receive psychological support, not any screening or diagnosis and that there is a potential of referral onwards if any concerns arise from the consultation. Confidentiality is also assured. In times of pandemic, it is not incumbent upon psychiatry to skip or neglect boundaries; instead, we need to find ways to adapt them to dynamic situations of contagion and consent our clients openly and firmly about the boundaries that need to be adhered to.

## Discussion

This paper outlines two ways UBPI is currently being utilised during the COVID-19 pandemic: i) a self-guided and peer-supported intervention for occupational mental health and ii) a virtual PFA to address anxiety and distress. Both uses of UBPI have been deployed within a limited resource setting. In the first use, there is a limitation of human resources; and the second use has a restriction in delivery capability. UBPI was aptly adapted to address both barriers. UBPI also allows previously complicated psychotherapy skills, requiring multiple sessions, to be delivered as a brief intervention within a much shorter duration. All of these increase the ability of UBPI to be delivered with ease and reach as many recipients as possible.

The availability of manuals for both public and occupational settings also allow the UBPI to be implemented in a more standardised manner across services. The manual was also created with a strong focus on containing the minimum essential skills for the techniques used, meaning that it is easy to use. The manual uses a flexible ‘toolkit’ approach, where practitioners can choose the most appropriate ‘tool’ for the situation and is licenced under a Creative Commons licence, meaning that other practitioners can use it freely and modify where appropriate. This makes the manual easy to adapt. These elements made training in UBPI more accessible, even amongst practitioners without prior psychological training. The manual can also be translated into other languages. This was done with the manual created explicitly for COVID-19 pandemic where the Malay language was used.

The main limitation and criticism for UBPI is the lack of quantitative evidence. UBPI is based primarily on the usage of the right technique in the appropriate context. While qualitative feedback on the use of UBPI had been positive, measuring it quantitatively has proven to be difficult. Each client of UBPI can present with a wide variety of different issues. Also, each psychological skill used has a different theoretical background and measurements. There are currently no single measurement tools that can encompass the variety of presentations and theoretical background of the psychological skills. One possibility of quantitative measures is to focus on a self-reported improvement scale. However, that measurement method has its own biases and limitations.

## Conclusion

UBPI has the potential to be adopted on a wider scale by healthcare at large. It has been used in two crisis situations with occupational mental health and PFA. Many scientific and social innovations have arisen out of wartime events or acute crisis situations, be it the recruitment of women into employment during the World War I, or advancement of anaesthesia and surgery techniques in World War II. COVID-19 has similar potential, like a pandemic of unforeseen proportions, causing corresponding anxiety and distress of potentially unpredictable proportions, to usher in an era of higher psychological sensitivity and broader provision of psychological interventions to those requiring all levels of support.

## Figures and Tables

**Table 1:** List of techniques adopted from other psychotherapies in UBPI

**ACT**	**MI**
Values and goals	Decision to change
Mindfulness	Problem solving
Physicalisation	
Experience avoidance	**EIP**
Anchoring	Care plan
	Staying well plan
**CBT**	Relapse prevention plan
Vicious cycles and triggers	Activity scheduling
Thought diary	
Mood diary	**Others**
	Therapeutic alliance
**DBT**	Shared decision making
Validation	
Boundary setting	
Autonomy supportive environment	
Assertiveness	
